# Editorial: Targeted drug delivery and mode of action of small molecules in neuroinflammation

**DOI:** 10.3389/fphar.2026.1782890

**Published:** 2026-02-12

**Authors:** Vishal S. Patil, Pukar Khanal, Darasaguppe R. Harish

**Affiliations:** 1 Department of Pharmacology, KLE College of Pharmacy, Belagavi, KLE Academy of Higher Education and Research, Deemed-to-be-University, Belagavi, Karnataka, India; 2 Department of Pharmacology and Chemical Biology, Emory University School of Medicine, Atlanta, GA, United States; 3 ICMR-National Institute of Traditional Medicine, Belagavi, Karnataka, India

**Keywords:** Alzheimer’s disease, drug discovery, nanoparticles, neuroinflammation, neurological disorders

## 1 Introduction

Alzheimer’s disease (AD), Parkinson’s disease (PD), and other dementia related metabolic disorders are linked to neuroinflammation as a key pathogenic process. Pro-inflammatory cytokines, chemokines, reactive oxygen species, and inflammasome are excessively released when microglia and astrocytes are activated. Although small molecules modulating these inflammatory cascades have a great deal of therapeutic promise, poor bioavailability, quick systemic clearance, off-target toxicity, and, most importantly, limited penetration across blood-brain barrier (BBB) continue to hinder their successful translation into effective brain therapies. Recent advances in computational and experimental biology, pharmacology, chemistry, and nanotechnology are being integrated to address these unmet clinical needs. It is feasible to develop treatments that are safer, more effective, and more in line with the intricate biology of neurodegenerative disorders by combining these delivery methods with mechanistic and systems-level analysis.

## 2 Nanocarrier-based strategies for brain-targeted drug delivery

Liposomes, polymeric nanoparticles, solid lipid nanoparticles, and dendrimers as nanocarrier systems have become innovative methods to overcome delivery hurdles associated with BBB. These allow small molecules to be encapsulated, improving their bioavailability and protecting from early breakdown. Crucially, nanocarriers regulate drug release, limit systemic exposure, and maintain therapeutic concentrations in brain regions. BBB traversal and site-specific drug accumulation have been enhanced by recent advancements in nanocarrier engineering, e.g., surface functionalization with targeting ligands, receptor-mediated transport methods, and stimuli-responsive release mechanisms. These methods enable nanocarriers to release their payload preferentially at injured areas in response to inflammatory milieu. Wang et al. highlighted nanocarriers as transformative platforms for AD therapy to overcome BBB constraints to target neuroinflammation driven by microglial/astrocyte activation, TNF-α/IL-1β/IL-6/ROS release, and Aβ/tau feedback loops. These facilitate ligand-mediated transcytosis, stimuli-responsive release, and increased bioavailability with low toxicity. It highlights translational research, e.g., immunogenicity, clearance, and scalability, and suggests biodegradable PLGA, theranostics, and biomarker-driven personalization (APOE4/CRP) combined with CRISPR/siRNA for precision targeting.

## 3 Experimental validation of neuroprotective and anti-inflammatory effects

To connect delivery innovation with therapeutic impact, experimental validation is necessary after drug formulation. Mechanistic proof of small-molecule activity is provided by *in vitro* tests evaluating oxidative stress attenuation, cytokine suppression, and inflammatory signaling pathway regulation. Pharmacokinetic and pharmacodynamic behavior in brain can be explained by *in vivo* employing mouse models of neuroinflammation. Morris water maze, rotarod, and sensorimotor tests correlate to molecular and cellular discoveries, connecting neuroinflammatory regulation in memory, learning, and motor coordination. In this Research Topic, Mujtaba et al. demonstrated intranasal chitosan nanoparticle (CSNP) *in situ* gels of escitalopram oxalate to enhance brain targeting and overcome oral bioavailability limitations. Optimized CSNPs showed sustained release, high entrapment, and improved brain pharmacokinetics, with Cmax and AUC significantly higher than oral solutions. These results highlight CSNP *in situ* gels as a promising nasal delivery system.

## 4 Computational approaches to elucidate mechanism of action

By identifying important biological targets, signaling hubs, and route linkages, network pharmacology emphasizes how effective neuroinflammatory therapies are inherently polypharmacological. Integrating research on neonate hypoxia-ischemia brain damage and AD, Zhang et al. identified shared hubs and inflammatory pathways. This shows related signaling networks can be modulated to influence disease progression, connecting bioinformatics insights with possible therapeutic interventions. Molecular docking and molecular dynamics simulations provide information on binding stability, conformational flexibility, and contact energetics under physiological conditions, and enable atomistic investigation of drug-receptor and drug-nanocarrier interactions. If paired with experimental validation, these computational frameworks speed up rational drug design, decrease trial-and-error testing, and improve formulation optimization.

## 5 Multi-targeting, drug repurposing, and translational opportunities

Given the multifactorial nature of neuroinflammation, therapeutic strategies targeting single pathways yield limited and transient benefits. Therefore, network pharmacology and systems biology increasingly support multi-targeting strategies. Small molecules capable of simultaneously modulating several inflammatory and neuroprotective pathways may more accurately reflect complex biology of neurodegenerative diseases. Natural small molecules originating from traditional medicine are valuable, as many show polypharmacological profiles aligned with neuroinflammation regulation. Drug repurposing strategies aim to identify existing small-molecule therapeutics with previously unrecognized anti-neuroinflammatory potential. Repurposing can minimize development timelines and accelerate clinical translation by leveraging established safety and pharmacokinetic profiles. In this Research Topic, Luo et al., in mouse models, showed marein confers neuroprotection in cerebral ischemia–reperfusion injury by reducing oxidative stress, and inhibiting pro-inflammatory cytokines. Similarly, Zhang et al. demonstrated calycosin protecting cerebral ischemia–reperfusion injury by inhibiting HMGB1–NLRP3–caspase-1–GSDMD–IL-1β/IL-18 pyroptosis signaling. Furthermore, Li et al. showed, in pseudo germ-free rats, that co-administration of Senkyunolide I with Chuanxiong enhances bioavailability and reduces neuroinflammatory responses, underscoring potential of combinatorial traditional compounds for neuroprotection. Wei et al. reported glycyrrhizin modulating pyroptosis, providing both neuroprotective and anticonvulsant effects. In addition, Zheng et al. used integrated computational target identification, molecular docking, and dynamics to identify monoamine oxidase A as nimodipine’s target, supporting its potential in neurodegenerative diseases and oxidative-stress modulation. Lee et al. further demonstrated FDA-approved FGFR inhibitor erdafitinib diminishes neuroinflammatory responses by suppressing NLRP3 inflammasome activation *in vitro* and *in vivo*. These studies illustrate how systems biology and network pharmacology, combined with multi-targeting small molecules, traditional medicine–derived compounds, and drug repurposing approaches, may accelerate translational progress, maximize therapeutic benefit, and address neuroinflammation.

## 6 Novel targets in neurological disorders

To develop next-generation therapeutics, it is essential to identify and characterize novel molecular targets. Chemokine axes, microglial receptors, pyroptosis regulators, and synaptic signaling nodes emerged as intriguing pathways for disease modification. In this Research Topic, CX3CL1/CX3CR1 axis function in AD is explained by Yang et al. To control glial activation and neuroinflammation in central nervous system, chemokine CX3CL1 interacts with CX3CR1. Although its effects on Aβ deposition are complex and may vary depending on disease stage, authors propose CX3CL1/CX3CR1 may be beneficial in AD by lowering neuroinflammation, neurotoxicity, and tau phosphorylation. Review emphasizes how CX3CL1/CX3CR1 may be used as therapeutic target for neuroprotection in AD and other neurodegenerative conditions.

## 7 Scope and outlook of research topic

Goal of this Research Topic is to explain the purpose of modulating neuroinflammation by addressing crucial problem of delivering small molecules to brain. To increase targeting accuracy, therapeutic efficacy, and safety, we aimed to emphasize interdisciplinary techniques that incorporate nanocarrier-based delivery systems, experimental validation, and computational modeling. Submissions integrating network pharmacology with *in vitro* and *in vivo* research were encouraged following the use of molecular dynamics simulations under physiologically relevant settings to validate compound-protein interactions. This Research Topic aims to enhance our mechanistic comprehension of small-molecule mechanisms of action in neuroinflammation by combining developments in nanotechnology, pharmacology, and computational science. At the end, these approaches will open door for next-generation treatments to handle clinical difficulties, complexity, and heterogeneity of neuroinflammatory and related neurodegenerative disorders.

